# Single-cell omics: A new direction for functional genetic research in human diseases and animal models

**DOI:** 10.3389/fgene.2022.1100016

**Published:** 2023-01-04

**Authors:** Siyuan Kong, Rongrong Li, Yunhan Tian, Yaqiu Zhang, Yuhui Lu, Qiaoer Ou, Peiwen Gao, Kui Li, Yubo Zhang

**Affiliations:** ^1^ Shenzhen Branch, Guangdong Laboratory for Lingnan Modern Agriculture, Key Laboratory of Livestock and Poultry Multi-omics of MARA, Animal Functional Genomics Group, Agricultural Genomics Institute at Shenzhen, Chinese Academy of Agricultural Sciences, Shenzhen, China; College of Animal Science and Technology, Qingdao Agricultural University, Qingdao, China; ^2^ Shenzhen Branch, Guangdong Laboratory for Lingnan Modern Agriculture, Key Laboratory of Livestock and Poultry Multi-omics of MARA, Animal Functional Genomics Group, Agricultural Genomics Institute at Shenzhen, Chinese Academy of Agricultural Sciences, Shenzhen, China; ^3^ College of Animal Science and Technology, Qingdao Agricultural University, Qingdao, China; ^4^ College of Life Science and Engineering, Foshan University, Foshan, China

**Keywords:** single-cell omics, multi-omics integration, human disease, cell lines, animal disease models

## Abstract

Over the past decade, with the development of high-throughput single-cell sequencing technology, single-cell omics has been emerged as a powerful tool to understand the molecular basis of cellular mechanisms and refine our knowledge of diverse cell states. They can reveal the heterogeneity at different genetic layers and elucidate their associations by multiple omics analysis, providing a more comprehensive genetic map of biological regulatory networks. In the post-GWAS era, the molecular biological mechanisms influencing human diseases will be further elucidated by single-cell omics. This review mainly summarizes the development and trend of single-cell omics. This involves single-cell omics technologies, single-cell multi-omics technologies, multiple omics data integration methods, applications in various human organs and diseases, classic laboratory cell lines, and animal disease models. The review will reveal some perspectives for elucidating human diseases and constructing animal models.

## 1 Introduction

### 1.1 The introduction of single-cell omics

**FIGURE 1:**
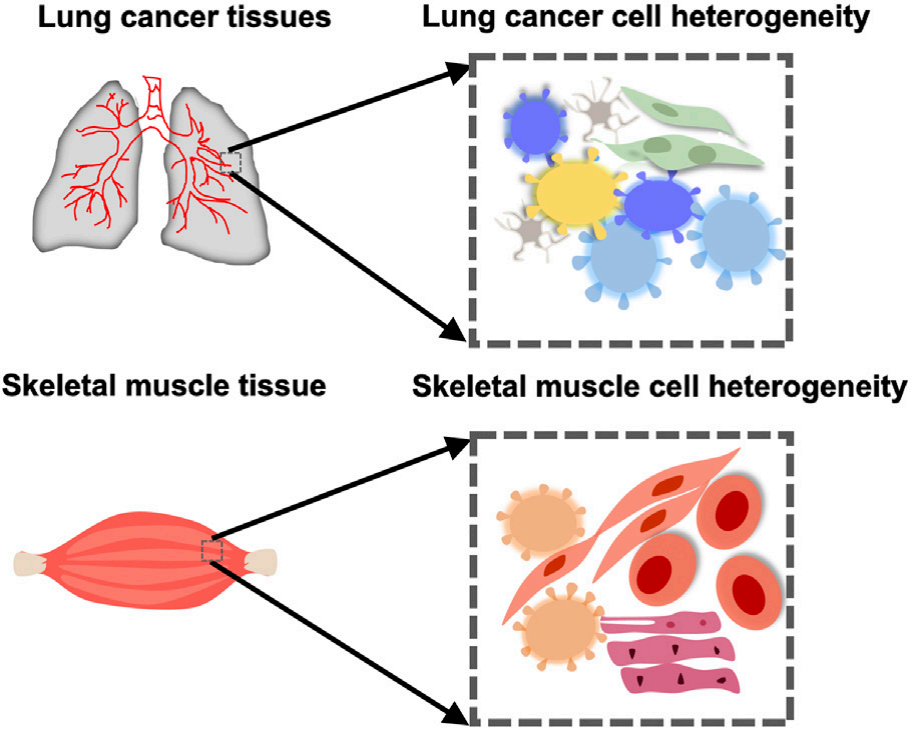
Cellular heterogeneity in an organ or tissue in health or cancer. Muscle cells take on different forms in the skeletal muscle. There is also heterogeneity between cells in the lung cancer tissue.

**FIGURE 2:**
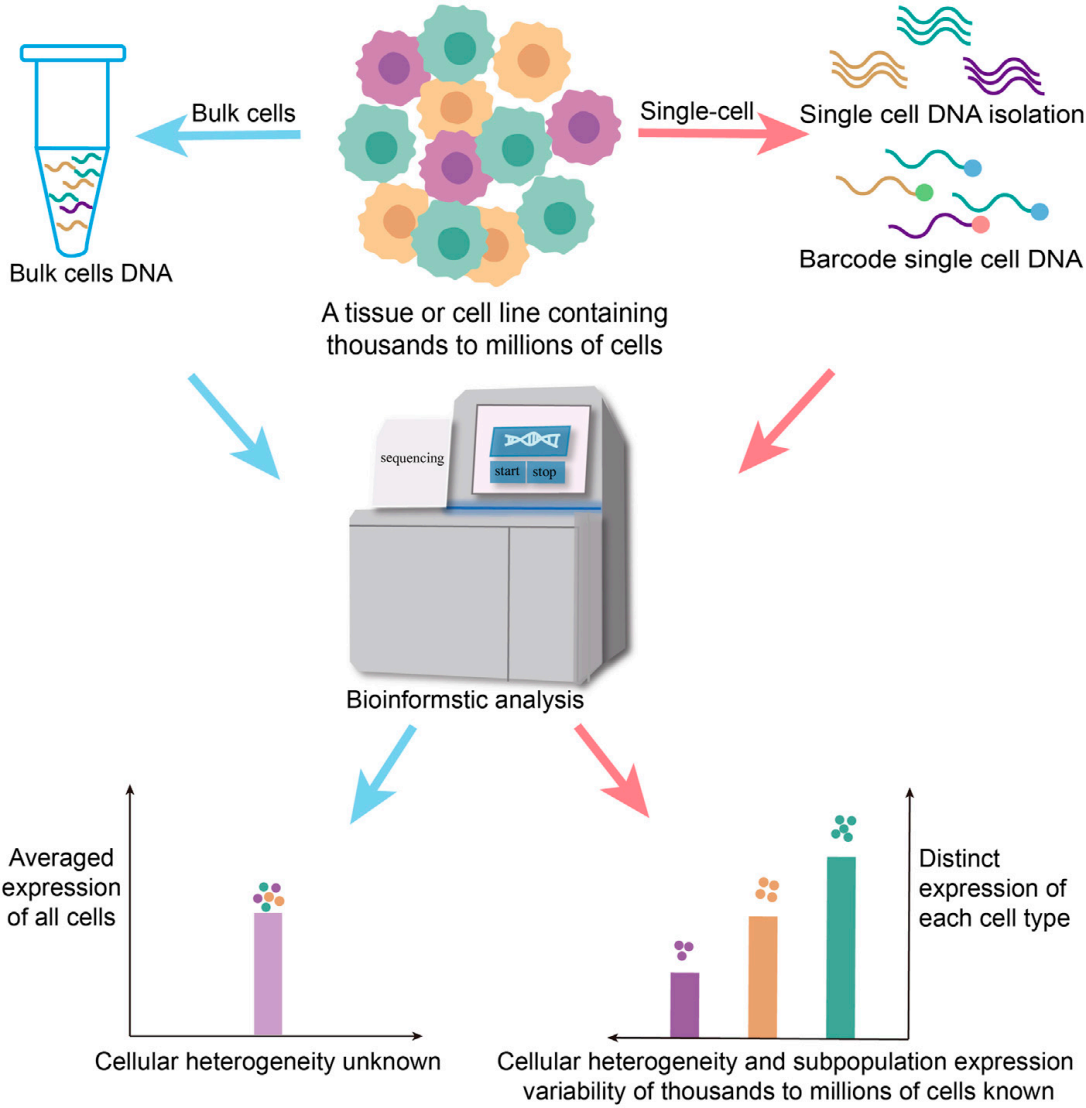
Traditional biological research is carried out from bulk cell levels, and the experimental results are often the averaged gene expression of the cell population, or only represent the life activity information of the dominant cell in the number and cannot accurately reflect a lot of information about cell heterogeneity in the sample. It ignores the differences in gene expression regulation between different population and single cells. Single-cell sequencing, which is sequenced at the individual cell level, solves the heterogeneity of genetic variation between different cells.

Cells are the structural and functional units of organisms. Traditional biological research is mostly at the population level, focusing on the average of the tissue/cell population but ignoring the characteristics of individual cells. Cells are heterogeneous, not only are there differences in cell phenotypes but also in their biological functions, such as transcriptional regulation, gene expression, and signal transduction (Wen and Tang, 2018a; Chappell et al., 2018). For example, the heterogeneity among malignant tumor cells is of great significance for analyzing the mutation mode, regional differences, evolutionary laws, and drug resistance mechanisms (Figure 1). Following the considerable development of single cell isolation and next-generation sequencing (NGS) (Mardis, 2011), single-cell transcriptome (Tang et al., 2009), genome (Navin et al., 2011), and methylation sequencing (Guo et al., 2013) have emerged. A state of the cell is determined by the dynamic states of its three-dimensional (3D) chromatin conformation, chromatin remodeling, regulatory element interaction, epigenetic modification, transcriptional regulation, gene expression, proteome, and metabolome (Chappell et al., 2018). These have also become the basis for single-cell research, revealing the diversity of cell types and subsets (Figure 2). Simultaneously, a series of single-cell mono-omic sequencing technologies were developed for the purpose.

These technologies reveal the heterogeneity of single cells on different levels. But each cell can only be used to reveal one kind of omic information. The main challenge in current single-cell omics is determining how to stimunously reveal various omic features within an individual cell. Until recently, single-cell multi-omics has been an effective solution. On the one hand, single-cell multi-omic intergrating analysis can combine many single-cell mono-omic sequencing data to elucidate life activities. On the other hand, they are able to obtain two or more types of omic data from one cell at the same time.

### 1.2 Development of single-cell omics

Single-cell transcriptomics can intuitively reflect heterogeneity and functional differences in expression levels and is often used as a referenceable single-cell omics technique (Efremova and Teichmann, 2020). In addition, molecular cellular identity is a product of the interplay between various modalities. It is essential to make coordinated measurements linking different regulatory layers to comprehensively understand how individual cells can demonstrate heterogeneity (Ogbeide et al., 2022). Parallel to the rapid and widespread adoption of transcriptomics, other single-cell omics technologies have been developed and evolved in a variety aspects within cells, including the genome (Gawad et al., 2016; Lan et al., 2017), methylome (Guo et al., 2013; Guo et al., 2014; Guo et al., 2015; Schwartzman and Tanay, 2015), histone modification (Park, 2009; Rotem et al., 2015), chromatin accessibility (Buenrostro et al., 2015a; Chen et al., 2018), chromatin conformation (Nagano et al., 2013; Nagano et al., 2015; Nagano et al., 2017), proteome (Shahi et al., 2017), nucleosome localization (Chereji et al., 2019), spatial transcriptome (Ståhl et al., 2016), metagenomic (Yilmaz and Singh, 2012) and even microbiome (Lloréns-Rico et al., 2022). Absorbingly, the development of single-cell omics has laid the foundation for capturing multiple omics in a single cell.

In addition to the multi-omics joint analysis of single cell omics, single-cell multi-omics has also developed into some high-order techniques such as single-cell two-omics and single-cell triomics in recent years. Single-cell multi-omics (≥2) technologies sequence and analyze multiple omics for single cells. In 2015, G&T-seq (genome and transcriptome sequencing) and DR-seq (gDNA-mRNA sequencing) have been developed for parallel measuring genomic and transcriptomic data, but the throughput is low (Dey et al., 2015; Macaulay et al., 2015). Excitingly, with the advent of 10×Genomics strategy in 2016, single-cell multi-omics technology is advancing rapidly. Following single-cell sequencing technology, which was named Nature’s 2013 Technology of the Year (Nature Methods, 2013), single-cell multi-omics became the Technology of the Year in 2019 (Nature Methods, 2019).

### 1.3 Studying single-cell omics for function genetic research in human and animal model

Over the past decade, genome-wide association studies (GWAS) have uncovered thousands of genes and genetic variants (SNPs) associated with human disease (Boyle et al., 2017). However, the biological mechanisms and functional features behind these associations have not yet been adequately mined (Klein et al., 2005; Gallagher and Chen-Plotkin, 2018). Most SNP loci are found in non-coding regions of the genome by whole genome sequencing and resequencing, which complicates understanding the pathogenic mechanisms of SNPs. Additionally, the transcriptome, metabolome, proteome, and phenome also provide rich information about phenotypic variation. Integrating GWAS data and phenotypic variation is an important step in studying pathogenesis. Generally, a large number of SNPs play roles at the tissue or cell-specific level. Due to the cellular heterogeneity of the focal organs, single cell resolution research is needed. Using the single-cell omic methodology, scientists could map disease-associated SNPs or genes to cell types at the single-cell level, reveal the heterogeneity among cells at multiple omic levels and elucidate their different omic associations or variations, which would provide new directions for functional genetic research in human diseases and animal models (Timshel et al., 2020; Tomaszewski et al., 2022).

Although there are many excellent reviews about single-cell sequencing (Nawy, 2014; Schwartzman and Tanay, 2015; Wang and Navin, 2015; Bian et al., 2018; Wen and Tang, 2018b; Kang et al., 2018; Tang et al., 2019; Zhu C. et al., 2020; He et al., 2020; Lee et al., 2020; Lloréns-Rico et al., 2022), we review the methodological progress and application of single-cell omics and single-cell multiomics, computational methods and tools for single-cell omics analysis, and the future research trend for analyzing the pathogenesis of human diseases and animal models in the post-GWAS era. The review tries to reveal some new ideas in analyzing disease mechanisms and constructing animal models of human diseases.

## 2 The methodological progress of single-cell omics and single-cell multi-omics

### 2.1 Single cell isolation

**FIGURE 3:**
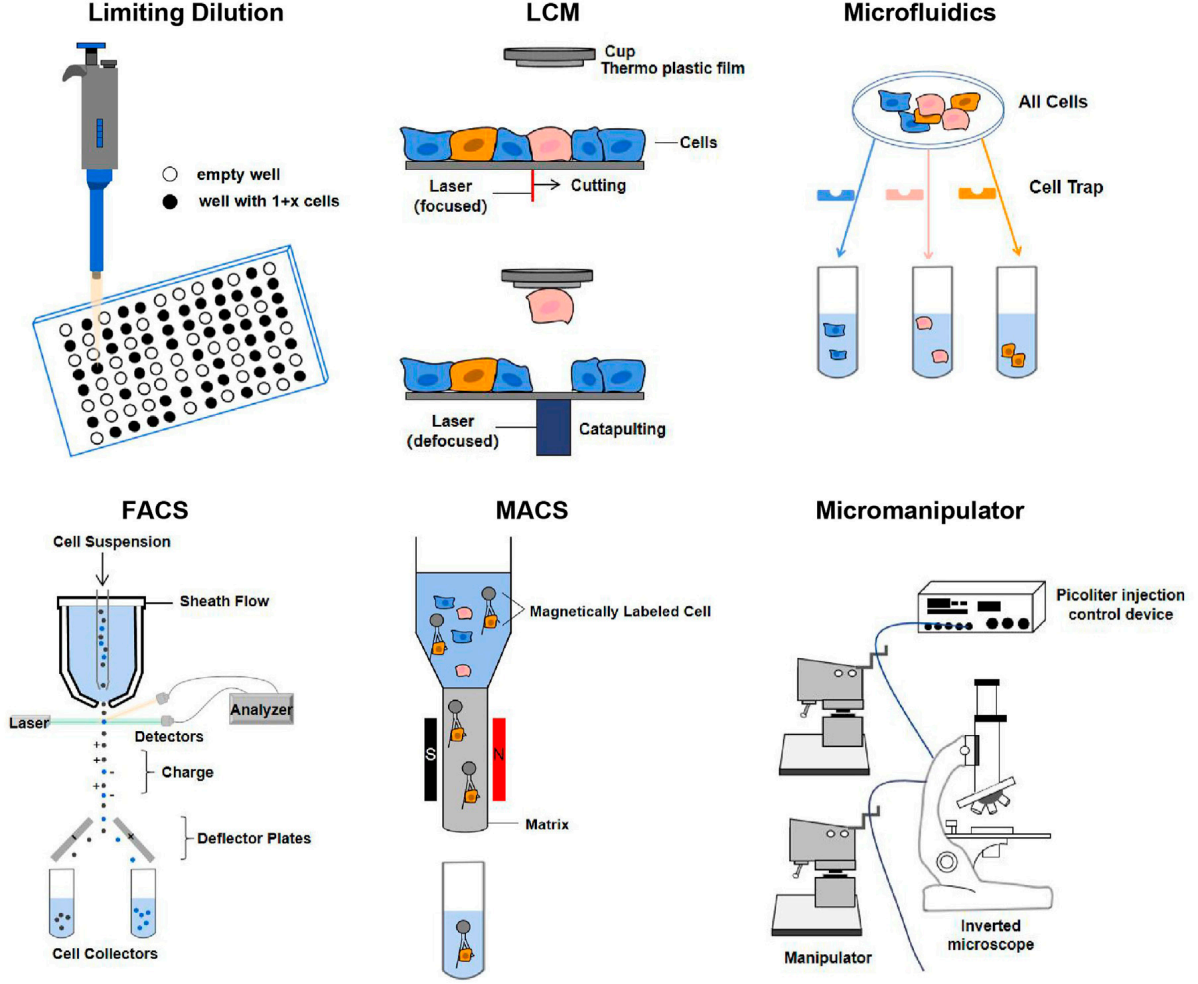
Single-cell isolation techniques. The principles of each technique are described in the text. Abbreviation: LCM, laser capture microdissection; FACS, fluorescence-activated cell sorting; MACS, magnetic-activated cell sorting (Gross et al., 2015; Hu et al., 2016).

Efficient isolation of single cells is a prerequisite for single-cell omics and multi-omics techniques. The quality of separation technology is mainly determined by its integrity, purity, quality, sensitivity, and throughput (Gross et al., 2015). Based on these requirements, a variety of different isolation methods have been developed, such as manual cell picking, limiting dilution, laser cell manipulation (LCM), microfluidics, fluorescence-activated cell sorting (FACS), and magnetic-activated cell sorting (MACS). They are illuminated in Figure 3.

Particularly, the application of cell barcoding with combinatorial indexing has gradually emerged in recent years. Cell barcoding is a single-cell tracking strategy, that is, capable of sequencing libraries from multiple cells (Cusanovich et al., 2015). FACS can use the combined index strategy to classify the cells in the plates using a unique barcode marker, so that each cell has a unique barcode to achieve the purpose of single cell separation. But combinatorial indexing methods can lead to the loss of many cells (Chappell et al., 2018). Except for the methods mentioned in Figure 3, other single-cell isolation techniques are comprehensively summarized in Supplementary Table S1.

Due to the technical limitations of preparing suspensions directly from fresh tissue to isolate intact single cells, it is challenging for researchers to analyze gene expression at the single-cell level. Interestingly, single-nucleus library construction sequencing technology was invented. For instance, sNuc-seq is capable of isolating a single nucleus, using it as the raw material, and resolving different cell types and dynamic changes (Grindberg et al., 2013).

### 2.2 Single-cell omics technologies

Here we briefly introduce the use and methodological development of multiplex single-cell mono-omics sequencing technologies.

#### 2.2.1 Single-cell genomics

The research of single-cell genetic information dates back to the 1970s in the field of cell and immunology, where cytologists used karyotyping, fluorescence *in situ* hybridization (FISH) and Giemsa staining to study genome rearrangement at the single-cell level. With the advent of PCR technology in the 1980s, scientists were able to directly amplify and sequence single-cell genomic DNA, but with limited coverage of the genome. The isolation of Phi29 (Φ29) and Bst polymerase brought single-cell genome research into the era of whole genome amplification (WGA) (Navin and Hicks, 2011). WGA combined with the probe-based array-based Comparative Genomic Hybridization (aCGH) method could detect single-cell genome copy number variation, but with low resolution and reproducibility, even high noise (Fiegler et al., 2007; Navin et al., 2011). Differs from aCGH technology, the NGS detects whole genome DNA in a continuous, long, non-targeted manner with the improved throughput and lower costs, has become a priority choice (Navin and Hicks, 2011). Combining WGA and NGS, single-cell whole genome sequencing (scWGS) can effectively reveal genetic variation in the genome of a single cell, such as copy number variation (CNV), single-nucleotide variants (SNV), and other structural variants (Navin et al., 2011). A variety of single-cell genome sequencing technologies have become powerful tools for detecting cellular heterogeneity, such as single-cell genomic sequencing (SIC-seq), single-cell combinatorial marker sequencing (SCI-seq), and topographic single cell sequencing (TSCS) (Navin et al., 2011; Vitak et al., 2017; Casasent et al., 2018). However, due to the short read limit of the second-generation sequencing platform, these methods cannot exhaustively detect genomic structural variation (including insertion, deletion, repetition, translocation, etc.) (Futreal et al., 2004). Interestingly, cytogenetic profiles are also of importance as the karyotype alterations (both structural and numerical) change the system information by creating new karyotype coding (Ye et al., 2019). Recently genome chaos, including chromothripsis, polyploidy giant cancer cells (PGCCs), and micronuclei clusters, has become a hot topic (Stephens et al., 2011; Liu et al., 2014; Niu et al., 2016), as they are essential for genome instability-mediated somatic evolution. Single-cell omics are needed to characterize these reorganized karyotypes. Methods are also required to convert the sequence data into cytogenetic data (Zhang and Kschischo, 2022). Tang et al. recently developed a single-cell genome sequencing method based on the third-generation sequencing (TGS) platform, called SMOOTH-seq (single-molecule real-time sequencing of long fragments amplified through transposon insertion). This method is a new breakthrough in scWGS by optimizing reaction conditions and linker sequences to obtain reliable and efficient single-cell SVs and ecDNAs with longer genomic readings (Fan et al., 2021). With the advantages of TGS platforms, this method has a wide range of application potential in the field of single-cell genomics. Moreover, we summarized as many single-cell genome sequencing techniques as possible in SupplementaryTable S2.

#### 2.2.2 Single-cell transcriptome sequencing

Initially, Tang et al. used poly (T) primers to reverse single-cell mRNA to obtain single-stranded cDNA, then added poly(A) to its end to obtain double-stranded cDNA, and finally used the Applied Biosystem sequencing platform SOLiD system to complete the first true single-cell transcriptome sequencing in 2009 (Tang et al., 2009). Subsequently, a large number of methods are built based on this principle (Supplementary Table S3) (Sasagawa et al., 2013; Fan et al., 2015; Nakamura et al., 2015; Sheng et al., 2017). Generally, single-cell RNA sequencing (scRNA seq) consists of four steps: 1) single cell isolation and lysis, 2) reversal to cDNA, 3) cDNA amplification, 4) library construction and sequencing.

Single-cell isolation for single-cell transcriptome sequencing has evolved from a single centrifuge tube, 96/384-well plates, or water droplets in oil. By adding cell identification code barcodes to the inverted primer or template switch oligo (TSO) primer to a mount of cells, improving the sequencing throughput and reproducibility. SCRB-seq (single-cell RNA barcoding sequencing) and mcSCRB-seq using this strategy of enriching 3′ ends with Barcode and UMI for high-throughput transcriptome sequencing based on higher-throughput 96/384-well plates (Soumillon et al., 2014; Bagnoli et al., 2018). But it is difficult to achieve a tens of thousands of cell count. Drop-seq is high-throughput method and isolates cells in oil-coated water droplets, using special magnetic beads with Barcode and UMI to grasp the polyA tail of mRNA, each primer on the beads grabs the mRNA of a single independent cell, and then collects these beads for inversion, template replacement, and amplification to constuct a library. But the equipment cost of additional droplet pump systems is higher (Macosko et al., 2015). Among 13 single-cell RNA sequencing methods, CEL-seq2, Quartz-seq2 and Smart-seq2 were found to have low-throughput, but the 10 x Chromium performing well in high-throughput (Mereu et al., 2020). At present, the 10x Genomics platform based on water-in-oil droplets combined with high-throughput single-cell sequencing is widely used. But the problem of probabilistic collision affecting the capture efficiency in 10x can’t be avoided. In 2018, Han et al. established the Microwell-seq method to capture mRNA in nanopores using magnetic beads (Han X. et al., 2018). Ensure that each microwell contains a cell and a magnetic bead by gradient dilution, and then grasp the mRNA after lysis of the cells in the microwell. Nevertheless, the beads used in the Indrop, Drop-seq, and Microwell-seq methods are synthesized using the split-and-pool principle, which has been commercially produced but is still expensive. Different from the above methods, in SPLiT-seq, each cell is labeled with a specific barcode combination label, increasing the throughput and reducing the cost (Rosenberg et al., 2018).

Next,for reversal to cDNA, the single-cell transcriptome library building method is developed according to the switching mechanism at the 5’ end of RNA template-based PCR (SMART). These include STRT-seq (Islam et al., 2012), Smart-seq (Ramsköld et al., 2012), Smart-seq2 (Picelli et al., 2014), Patch-seq and so on. SMART-seq2 technology is based on STRT-seq, but adds betaine to the reaction system to improve the thermal stability and reversal efficiency of the enzyme, while adding Mg2+ to combine betaine carboxylate anions to form an ionic pair that becomes a DNA instabilitizer. Then, using additional thermal cycling (50°C for 2 min; 42°C for 2 min) to unlock the secondary structure of RNA, improve the specificity of hybridization, and promote template conversion. At present, SMART-seq2, which has been optimized to achieve high sensitivity and high gene coverage, has become the gold standard for single-cell transcriptome sequencing and has been widely used in biological research.

However, because of the non-linear amplification caused by PCR, PCR-dependent transcriptome studies had a significant bias for quantitative analysis of RNA expression. *In vitro* transcription (IVT)-based linear amplification has been preferred in recent years, which can greatly reduce amplification bias. Such methods include CEL-seq (Hashimshony et al., 2012), CEL-seq2 (Hashimshony et al., 2016), MARS-seq (Jaitin et al., 2014), inDrops (Klein et al., 2015), etc.

Traditionally, scRNA-seq is limited to fresh and frozen samples, which could lose cell types and lead to inadequate dissociation or transcriptional stress responses due to digestion restrictions on the tissue. single-nucleus RNA sequencing (snRNA-seq) isolates individual nuclei then generates transcriptome information from isolated nuclei, which can avoid these problems to some extent,is suitable for cryopreserved or difficult-to-isolate tissues (Lake et al., 2016; Kim et al., 2018; Wu et al., 2019; Cui et al., 2020; Koenitzer et al., 2020; Slyper et al., 2020; Zhou et al., 2020; Richter et al., 2021). However, snRNA-seq requires lysing cells, which impedes further molecular or functional analyses of the same cells. Recently, Chen’s team developed a technology termed “live cell transcriptome sequencing technology” (Live-seq), which has achieved the survival and function of cells after single-cell transcriptome sequencing, so that the dynamic changes of cells can be tracked by minimally invasive extraction of cytoplasm in living cells and the expansion of extremely trace amounts of cytoplasmic RNA. This technology, which realizes the continuous observation of whole genome expression in living cells for the first time, provides a new research strategy for single-cell transcriptome sequencing (Chen et al., 2022).

#### 2.2.3 Single-cell epigenome

Single-cell epigenomes have the potential to provide a deeper understanding of cell type-specific gene regulatory procedures and how they change during development in response to environmental cues and disease pathogenesis. The current experimental single-cell platforms for analyzing different epigenomic features focus on DNA modification, histone modification, DNA-protein interaction, chromatin accessibility, and 3D chromatin conformation (Kong et al., 2020a; Preissl et al., 2022).

##### 2.2.3.1 DNA modification

The identity and function of different cell types are determined by the epigenome of the cell—a collection of covalent modifications of DNA and histones. The epigenome plays an important role in growth, development, and disease onset (Wen and Tang, 2018b).

For DNA methylation modification research, Guo et al. (2013) describe a methylation group analysis technique capable of performing single-cell and single-base resolution DNA methylation, named single-cell reduced-representation bisulfite sequencing (scRRBS). The technology is highly sensitive and can detect the methylation status of up to 1.5 million CpG sites in the genome of a single mouse embryonic stem cell (mESC). scRRBS can cover on average 70% of the CpG islands of the genome, which is characterized by small sequencing depth, low cost, and wide coverage. The single-cell bisulfite sequencing (scBS-seq) undergoes 5’ pre-amplifications to cover 3.7 million CpG sites, making it suitable for obtaining as many methylation sites as possible from single cells (Smallwood et al., 2014; Clark et al., 2017). However, the throughput of the above method is not high. The single-nucleus methylcytosine sequencing (snmC-seq) combines FACS and plate-based bisulfite treatment for increasing throughput (Luo et al., 2017). Mulqueen et al. (2017) describe a single-cell combinatorial indexing strategy (sci-MET) for methylation analysis that also significantly improves throughput (Mulqueen et al., 2017).

The process of demethylation produces a series of intermediate products, such as 5-hydroxylmethylcytosine (5 hmC), 5-formylcytosine (5 fC) and 5-carboxylcytosine (5caC). Identifying and studying these intermediates provides clues to DNA modifications, but the number of these oxidative derivatives is so small that traditional bisulfite sequencing cannot be distinguished, so special sequencing methods need to be developed. Mooijman et al. (2016) developed the scAba-seq method to locate 5 hmC The method is based on a special restriction enzyme, AbaSI, which recognizes glycosylated 5 hmC sites and produces double-strand breaks of double nucleotides 11–13 bp downstream of the 5 hmC sites. Similar to CEL-seq, scAba-seq introduces T7 promoter joints to conduct *in vitro* transcription. The library construction of ScAba-seq is based on barcode technology, which has significantly increased the throughput for single-cell analysis. By using it, 44,000 different 5 hmC sites have been identified in mouse ESCs. In 2017, Zhu et al. established chemical labeling-enabled C-to-T conversion sequencing (CLEVER-seq) for the analysis of 5 fC in single cells (Zhu et al., 2017). This method utilizes selective 1,3-indandione-mediated Friedländer labeling, similar to the bisulfite-free detection method (fC-CET), to induce 5 fC in PCR amplification and sequencing. Using this method, 3500 5 fCpG loci have been detected in mouse ESCs. Wu et al. (2017) report single-cell methylase-assisted bisulfite sequencing (scMAB-seq) for analyzing 5fC/5caC. CpG methyltransferase M. SssI was added before bisulfite conversion. M. SssI treatment converts unmodified cytosine in the CpG environment, followed by sequencing of 5 fC and 5caC sites read as T, and C/5mC/5 hmC sites read as C. Combining single-cell RRBS or Post Bisulfite Adapter Tagging (PBAT), the MAB-seq method is suitable for single-cell analysis of 5fC/5caC.

##### 2.2.3.2 Histone modification and DNA-protein interaction pattern

Genome-wide histone modifications are often positioned by chromatin immunoprecipitation and high-throughput sequencing techniques (ChIP-seq), which can enrich target chromatin fragments carrying specific histone modifications (Kong et al., 2022). ChIP-seq is also commonly used to analyze transcription factor binding sites and other protein-DNA interactions (Wen and Tang, 2018b). For example, Rotem et al. (2015) established the DropChIP method, which combines drop-based microfluidics and DNA barcoding to pool thousands of single cells prior to antibody immunoprecipitation, overcoming antibody limitations and increasing throughput, allowing nearly 1,000 modified sites to be detected at once.

##### 2.2.3.3 Open accessibility of chromatin

Gene regulation is affected by open chromatin accessibility. Open chromatin conformations are generally considered to bind more easily to *in vitro* enzymes. Buenrostro et al. (2015b) established a programmable microfluidic platform that can detect about 5,000 DNaseI hypersensitive sites (DHSs) at a time, with greater efficiency but a throughput of only 96. Meanwhile, the single-cell DNase-seq method developed by Jin et al. (2015) makes it possible to recover approximately 38,000 DHSs from single cells. The single-cell combinatorial indexed ATAC-seq (sciATAC-seq) is a strategy that uses combinatorial tags and Tn5 fragmentation, and was successfully used to study the genome-wide chromatin accessibility landscape for each of more than 15,000 single cells (Cusanovich et al., 2015). In 2018, Zhang’s team developed scTHS-seq technology to study epigenetic traits (Lake et al., 2018). This technique has some advantages, such as linear transcriptional amplification *in vitro* and the modified supermutant Tn5 transposase, which is more sensitive than scATAC-seq and improves the coverage of cell-specific distal enhancers.

Different from Dnase-seq and ATAC-Seq analysis, Nucleosome Occupancy and Methylome Sequencing (NOMe-seq) can provide more epigenetic information, because of its high resolution (25 bp). NOMe-seq could detect the chromatin status and DNA methylation at the same time. Scientists have developed a single-cell NOMe-seq technology called scNOMe-seq (Pott, 2017), as well as a few multi-omics methods, such as single-cell chromatin overall omic-scale land-scape sequencing (scCOOL-seq) (Guo et al., 2017) and single-cell nucleosome, methylation and transcription sequencing (scNMT-seq) (Clark et al., 2018).

##### 2.2.3.4 3D genomic conformation

Hi-C is the most widely used technique for deciphering gene regulation and cell function from the genomic 3D interactome. Single-cell Hi-C data can also be used to reconfigure the 3D genomic structure of individual cells, including A/B compartments, topologically associating domains (TADs), DNA-loops, etc. (Kong and Zhang, 2019; Kong et al., 2020b). In 2013, Nagano et al. used an intranuclear Hi-C strategy for single-cell Hi-C sequencing, performing chromatin crosslinking, restriction endonuclease cleavage, biotin filling, and ligation in permeated nuclei. It was able to detect 40,000–120,000 contacts in single cells (Nagano et al., 2013; Wen and Tang, 2018b). In 2017, Flyamer further simplified single-cell Hi-C to generate a snHi-C (single-nucleus Hi-C), which uses Phi29 whole genome amplification after chromatin crosslinking, DpnII digestion, and proximal ligation, while omitting biotin-related steps, enabling the detection of 400,000 contacts in single cells (Flyamer et al., 2017). For enhancing the throughput, Ramani et al. established a high-throughput single-cell combinatorial indexed Hi-C (sciHi-C) detection method using a combination labeling strategy (Ramani et al., 2017). The method is similar to sciATAC-seq and sci-MET, first combining the first round of barcodes with proximity ligation, and then combining the second round of barcodes through joint Y-adapter connections. The number of contacts detected can reach 9 times that of the original Hi-C.

However, to achieve high resolution of Hi-C analysis at the loop level, it is typically required to perform ultra-high-depth sequencing reads. However, it is easy to reach sequencing saturation, and the signals of interaction matrices are still sparse. Thus, Zhang’s team developed the DeepLoop tool on the basis of HiCorr to solve the problems of data sparsity and the sequencing bottleneck at kb resolution (Zhang et al., 2022). DeepLoop can identify high-resolution 3D genomic interactions from Hi-C data at very low sequencing depths, greatly reduces the required sample size and sequencing costs, realizing the low cost of high-resolution Hi-C analysis, and expands the Hi-C application in dynamic and single-cell 3D genome research.

Finally, single-cell epigenomic sequencing technologies are comprehensively described in Supplementary Table S4.

#### 2.2.4 Single-cell proteome

Because the protein content of a single cell is generally less than 200 pg and the complexity is high, the single-cell omics development of protein levels is very limited. The advent of Single Cell ProtEomics by Mass Spectrometry (SCoPE-MS) technology is particularly important for addressing the challenges faced by single-cell proteomes. SCoPE-MS delivers the proteome to the mass spectrometer with minimal protein loss, as well as simultaneously identifying and quantifying peptides in individual cells. This technique can detect more than 1,000 proteins in a single cell (Budnik et al., 2018). There is also another method for detecting cellular proteins, that uses antibodies that bind to DNA barcodes, measuring along with the transcriptomes of individual cells in the modified scRNA-seq method (He et al., 2020).

#### 2.2.5 Single cell metabolome

Single-cell metabolomics techniques are still in their infancy, and current methods have very limited sensitivity and considerable technical noise. Current extensions and improvements based on mass spectrometry methods have been able to detect small amounts of metabolites present in single cells (Comi et al., 2017; Zhang and Vertes, 2018; Zhu et al., 2018; Duncan et al., 2019).

#### 2.2.6 Single-cell microbiome

Individual microbial cells are highly heterogeneous, making it impossible for traditional omic techniques to distinguish the monas and verify the function of individual microorganisms. Some low-abundance communities are always ignored. The intergated use of both single-cell genomics and bulk metagenomics would efficiently obtain comprehensive and accurate genome-wide information from complex microbial communities. In recent years, some flagship technologies have been published to construct the single-cell microbiome.

##### 2.2.6.1 Microbial single-cell genome

The SiC-seq was developed and improved from single-cell genomics, is widely used for sequencing single-cell microorganisms in marine microbial samples (Lan et al., 2017). This method encapsulates individual cells in molten agarose droplets, polymerizes them to provide a semi-permeable matrix to fix bacterial cells, and then processes these microgels in a microfluidic device to generate a single-cell genome library for sequencing. Further, the researchers developed the SAG-gel method, which replaced the microfluidic processing step with sorting agarose droplets in microplates by flow cytometry sorting. For individual cells on microplates, genomic amplification is conducted. This technique has been applied to study the intestinal microbiome of mice, and 356 single amplified genomes (SAGs) have been successfully obtained (Chijiiwa et al., 2020). It is also widely used to obtain bacteria from the human mouth or intestine (SAGs). Excitingly, the recently developed Microbe-seq technique packages individual bacteria in water-in-oil droplets containing lysate. The droplets are genetically amplified after a series of mergers with other reagents in a microfluidic system (Zheng et al., 2017). This method has been used to analyze human stool samples, which could generate thousands of SAGs per sample. Microbial single-cell genome sequencing improves metagenomic genome assembly, resulting a greater biodiveristy of single microorganism genomes.

##### 2.2.6.2 Microbial single-cell transcriptome

scRNA-seq has been widely used to analyze cell types and states in eukaryotes, but existing methods are not applicable to microbes. To address this issue, Kuchina et al. (2021) developed microSPLiT (Microbial split-pool ligation transcriptomics), a low-cost, high-throughput method tailored to microbes. This technique enables comprehensive analysis of the transcriptome of thousands of cells, identifying rare or new subpopulations. It can be used to map dynamic gene expression changes in *Bacillus subtilis* at different growth stages.

### 2.3 Techniques for single-cell multi-omics

#### 2.3.1 Dual-omics in single cells

Different from single-cell monomic methods to obtain one-time omics data, single-cell multi-omics sequencing technology can simultaneously obtain multiple omics data from one single cell, which better reflects the association among different omics in a specific state and reveals a more “real” molecular regulatory network. For example, “single-cell dual-omics” refers to a single-cell library construction experiment achieving two omics sequencing at the same time (Macaulay et al., 2015).

Single-cell dual-omics sequencing techniques that combine single-cell transcriptomes and genomes were first used. These methods can analyze not only genomic differences and gene expression heterogeneity among cells, but also the relationship between genomic sequence differences, copy number variations, and transcriptome heterogeneity. TARGET-seq (Rodriguez-Meira et al., 2019), DR-seq (Dey et al., 2015), G&T-seq (Macaulay et al., 2015), SCGT (Li et al., 2015) and SIDR (Han K. Y. et al., 2018) are examples of such technologies. Subsequently, dual-omics sequencing techniques for simultaneous measurement of the transcriptome and epigenome in the same single cell have emerged to explore the epigenetic regulation of gene expression. For exploring the relationship between the transcriptome and DNA methylation, the methods include scMT-seq (Hu et al., 2016), scM&T-seq (Angermueller et al., 2016) and sc-GEM (Cheow et al., 2016). For combining transcriptome and chromatin accessibility, there are sci-CAR-seq (Cao et al., 2018), SNARE-seq (Chen et al., 2019), scCAT-seq (Liu L. et al., 2019), ATAC- RNA-seq (Hendrickson et al., 2018) and Paired-seq (Zhu et al., 2019). In detail, Liu et al. used scCAT-seq technology to study the regulatory relationship between cis regulatory elements and target gene expression, and assist in the diagnosis of embryonic quality before implantation in human embryos and cancer targets. There are also some technologies that combine transcriptomes with cell surface protein assays to identify relationships between different cell types and their cell functions, such as CITE-seq (Stoeckius et al., 2017), REAP-seq (Peterson et al., 2017), PLAYR (Frei et al., 2016) and PEA/STA (Genshaft et al., 2016). In complex tissues, cell spatial location and microenvironment also have important implications for cell function. Technologies such as Slide-seq (Rodriques et al., 2019), MERFISH (Moffitt and Zhuang, 2016; Travaglini et al., 2020), seqFISH+ (Eng et al., 2019), ST-RNA-seq (Giacomello et al., 2017), STAR-map (Wang X. et al., 2018), and osmFISH (Codeluppi et al., 2018) are capable of preserving cell spatial information while performing transcriptome sequencing to link cell function to the microenvironment. For instance, SeqFISH+ is based on the spatial multimodal approach for exploring nuclear tissue and cellular states applied to different biological systems (Takei et al., 2021). Moreover, Methyl-HiC (Li et al., 2019) and sn-m3C-seq (Lee et al., 2019) can simultaneously analyze DNA methylation and chromatin conformation, resolving the heterogeneity at these two aspects among different cells.

In addition to molecular detection between multiple omics layers, the combination of the gene editing technology CRISPR and single-cell sequencing to perform cell phenotype assays while exploring the effects of perturbation on gene regulatory networks deeply explores the causal relationship between gene expression regulation, cell function, and destiny (Jaitin et al., 2016; Eisenstein, 2020). In 2016, Jaitin et al. (2016) first used CRISP-seq to reconstruct the core molecular regulatory networks during differentiation and their stress response to pathogen infestation in blood medullary cells. Additionally, Perturb-seq (Adamson et al., 2016), CROP-seq (Datlinger et al., 2017), and Mosaic-seq (Xie et al., 2017) are examples of dual-omics sequencing techniques capable of detecting transcriptome and DNA perturbation simultaneously. In detail, the CROP-seq (CRISPR droplet sequencing) method optimizes CRISPR screening for analyzing thousands of gene editing events in single cells with high throughput and achieving a more detailed gene regulation analysis. In addition, Perturb-ATAC used the CRISPR library to detect chromatin accessibility, meanwhile, it removed trans regulators to study the effects of them on different cell regulatory networks (Rubin et al., 2019). Therefore, this technique could be used to identify the effects of transcription factors (TFs), chromatin-modifying factors, and non-coding RNAs (ncRNAs) on cell function maintenance and fate (Rubin et al., 2019).

#### 2.3.2 Triple-omics and multi-omics in single cells

Dual-omics sequencing technologies are also developing in the direction of more omics (for example, triple, quadruple, quintuple, and multi-omics). In detail, single-cell triple omics technology refers to a single-cell library building experiment to achieve three kinds of omics sequencing (Hou et al., 2016).

These multi-omics technologies include, for example, 1) Transcriptome + DNA copy number + DNA methylation triple-omics, scTrio-seq (Hou et al., 2016) and scTrio-seq2 (Bian et al., 2018), which have been used to study cancer occurrence and development mechanism. 2) The triple-omics of transcriptome + DNA methylation + chromatin accessibility, which includes scNMT-seq (Clark et al., 2018) and scCharM-seq (Yan et al., 2021). 3) Transcriptome + surface protein + CRISPR perturbation + TCR (T-cell receptor) cloning detection quadruple-omics: ECCITE-seq has been used to identify immune cell types (Mimitou et al., 2019; Overall et al., 2020). 4) Transcriptome + DNA copy number + DNA methylation + chromatin accessibility + chromosomal ploidy quintuple-omics: There are scCOOL-seq (Guo et al., 2017), iscCOOL-seq and scCOOL-seq2 (Gu et al., 2019). Tang et al. used the scCOOL-seq method to analyze the reprogramming of chromatin status and DNA methylation in mouse preimplantation embryos (Guo et al., 2017). These single-cell quintuple omics data can now be obtained from a single cell. With these, single-cell chromatin accessibility and DNA methylation can be investigated at single-base resolution, which greatly advances the ability to analyze the complex relationships among different genetic and epigenetic layers. Finally, multi-omics single-cell methods are comprehensively described in Supplementary Table S5.

### 2.4 Single-cell omics computational tools

Due to the development of single-cell omics techniques, how to integrate a large amount of different types of omics information is becoming a key problem, so the demand for single-cell omics computational analysis methods and tools is increasing (Hao et al., 2021). Efremova and Teichmann summarized computational methods for analyzing and integrating single-cell omics data across different patterns and discussed their applications, challenges, and future directions (Efremova and Teichmann, 2020). “Awesome Single Cell” (go.nature.com/2rmb1hp) on GitHub lists more than 70 tools and resources with a user-friendly interface for analyzing and integrating various single-cell sequencing data. Some methods and tools used to analyze single-cell omes have also been reviewed elsewhere (Stuart and Satija, 2019; Ma et al., 2020; Li et al., 2021; Miao et al., 2021).

Multi-omics data, including measurements on the same cell (matched data) and different cells (unmatched data). The matched multi-modal technology is the joining of different omics data by one sequencing technology on the same cell, such as sci-CAR, SNARE-seq, paired-seq, CITE-seq and REAP-seq. Many methods have been built for integrating matched multi-model data based on the three following approaches. A simple approach is to transform the data in such a way that all the measured attributes have homogeneous statistical characteristics. Traditionally, each feature is measured by the variation between samples. A more reasonable approach would be to give a probability score for each value of a feature, possibly using a different model for the feature set, so that the values can have a consistent probabilistic interpretation. Another more model-based theoretical method, is that which considers each omics-data as a “view” of the underlying relationship between cells. Tools using this approach include single cell Aggregation and Integration (scAI) (Jin et al., 2020), totalVI (Gayoso et al., 2021), multi-omics Factor Analysis (MOFA) and its later version, MOFA+ (Argelaguet et al., 2020). Unlike inferring a common representation space from multiple omics datasets, a type of late integration approach integrates data into the level of the inferred model, such as affinity in each modality.

**FIGURE 4:**
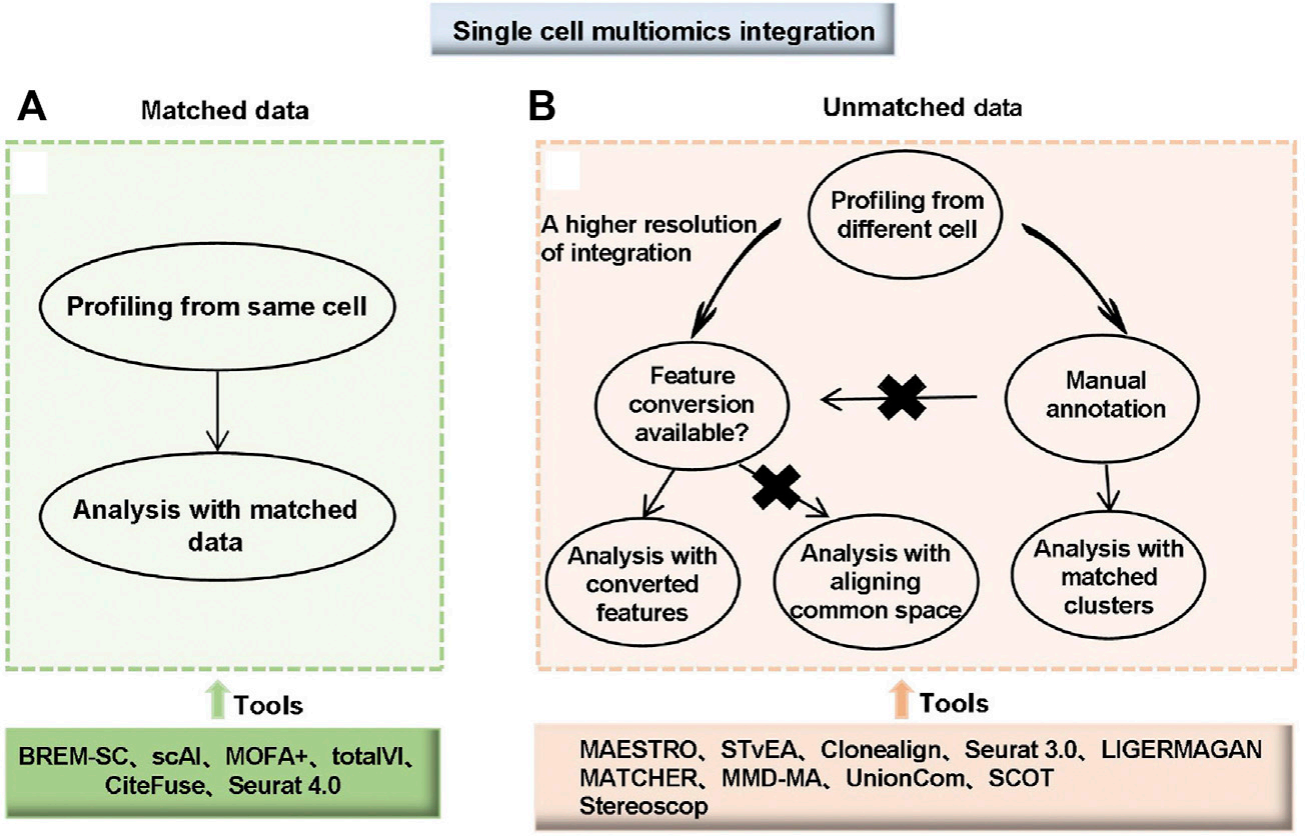
Choice and workflow of integrating single-cell multi-omics data. **(A)** Matched data means multi-omics data was measured from the same cell. **(B)** Unmatched data means multi-omics data was measured from different cells. For unmatched data, analyses can be performed with matched clusters if manual annotations of cell types are available. If manual annotations are not available or a higher resolution of integration is needed, two different strategies are available depending on whether feature conversion is possible. For data with a common feature set or converted features, tools developed for matching with converted features can be used. For data without common features or feature conversion, integration by aligning common spaces can be applied (Miao et al., 2021).

Unmatched data is measured on a different cell and faces one challenge: mapping the measurements from one modality to another. One integration approach is to match groups of cells between the modalities. For example, correlating clusters in each modality manually correspond to known cell types and other features with biological information, such as proximity of open chromatin to expressed genes. Second, match features based on their common molecular basis. For example, the STvEA tool, uses protein abundance as a common factor to match (Govek et al., 2021). Clonealign (Campbell et al., 2019) and Seurat 3.0 (Stuart et al., 2019) are based on some biologically motivated statistical models. When achieving a shared feature set through mapping among modalities was challenging or impractical, modeling the entire “space” of data by considering each mode and mapping these spaces to each other was an approach. This type of tool includes MATCHER (Welch et al., 2017), MMD-MA (Liu J. et al., 2019), UnionCom (Cao K et al., 2020) and SCOT (Demetci et al., 2020). Many integrative algorithms and analytical tools for scMulti-omics data are summarized in Supplementary Tables S6, S7; Figure 4.

## 3 The application of single-cell omics

### 3.1 Application and discovery in human organ/tissue development and disease

For organ/tissue development, the construction of the human cell map is fundamental to understanding the function of organs (Li et al., 2018; Wagner and Klein, 2020). The “International Human Cell Atlas” was planned to sequence the (about 37 trillion) cells in the human body, to dissect the composition, cell trajectory, function, and disease of human organs at the single-cell level (Regev et al., 2017). Petropoulos et al. isolated 1529 single cells from 88 pre-implantation embryos and mapped a complete transcriptional map for ascertaining the pre-implantation development of human embryos, revealing an intermediate state of cells in lineage-specific gene co-expression (Petropoulos et al., 2016). Using single-cell transcriptome sequencing technology, researchers from multiple institutions have analyzed the molecular mechanisms of human brain development and the association between genes and neurological diseases, such as autism, schizophrenia, and bipolar disorder (Wang D. et al., 2018). In addition to normal tissues and organs, transcriptome techniques can also be used to map organ disease states and cancerous cells. For example, Ordovas-Montanes et al. conducted a transcriptome profile analysis of respiratory epithelial cells, immune cells, stromal cells and their subsets in human type II inflammatory diseases by sequencing large-scale parallel single-cell RNA from samples of primary chronic sinusitis (Ordovas-Montanes et al., 2018). Lambrechts et al. measured transcriptome information from 52,698 cells in the human lung cancer microenvironment to identify 52 stromal cell subtypes and depict a complete lung cancer cell map (Lambrechts et al., 2018).

The single-cell omics platform provides a more comprehensive and clear picture of aging-related research (He et al., 2020). Multiple risk genes associated with Parkinson’s disease, amyotrophic lateral sclerosis, and schizophrenia have been identified through transcriptomics analysis (Olah et al., 2018). Both the single-cell genome and scRNA-seq demonstrate that somatic mutations accumulate with age and disease (Lodato et al., 2018; Zhang et al., 2019). Chromatin modification analysis also discovered that as people and cells aged, the variation of chromatin markers increased, resulting in a loss of transcriptional regulation (Cheung et al., 2018).

Single-cell omics has some applications in pre-implantation development, brain science, cancer tumor heterogeneity, tumor immunology, tumor resistance, and drug development (Liu J. et al., 2021; Nam et al., 2021). There are some examples of genetic disorders or genetic essentialities in discussions of human disease, there are some examples. Primary central nervous system lymphoma (PCNSL) is a rare form of central nervous system lymphoma. Limited material from CNS biopsies prevent a thorough characterization of PCNSL. By using single-cell RNA sequencing, B-cell receptor sequencing of rare PCNSL cells, and spatial transcriptomics of biopsy samples, results found that malignant B-cell in PCNSL exhibit transcriptional and spatial intratumor heterogeneity. T-cell exhaustion is common in the PCNSL microenvironment, where it co-localizes with malignant cells, highlighting the potential for personalized treatments (Heming et al., 2022). For molecular driver evolution, researchers decipher the intra-tumor and inter-lesion diversity of CTCL patients and propose a multi-step tumor evolution model using single-cell RNA analysis and bulk whole-exome sequencing on 19 skin lesions from 15 CTCL patients. They also establish a subtyping scheme based on the molecular features of malignant T-cell and their pro-tumorigenic microenvironments. These findings lay a strong foundation for comprehending the characteristics of CTCL and open the door to future precision medicine for patients (Liu et al., 2022). In the future, it will be more widely used in human growth, development, and tumor research, with the development of reproductive biology, developmental biology, and precision medicine.

### 3.2 Application in normal or disease cell lines

In biological and medical research, cell lines are often used as readily available materials or model cells. They are widely used and play important roles. The famous cell lines include K562 (lymphoblast cells isolated from the human bone marrow), HG002 (NA24385 human cell line), ESC (embryonic stem cells), C2C12 (murine premyoblast cell line) and HEK293T (human embryonic kidney cells). In this part, these cell lines are listed as examples to show the application. Genome assembly benefits from long-read sequencing technology with greater accuracy and continuity. However, because cellular heterogeneity can seriously affect haplotype assembly results, most current human genome assembly requires a large amount of DNA from homogeneous cell lines without maintaining cellular heterogeneity. Recently, the Tang team sequenced K562 and HG002 cells and assembled the *de novo* human genome on the PacBio HiFi and Oxford Nanopore Technologies (ONT) platforms using SMOOTH-seq (Xie et al., 2022). The study pioneered the assembly of the human genome with high continuity at the single-cell level (using 95 individual K562 cells, N50 is about 2 Mb) and explored the effects of different assemblers and sequencing strategies on genome assembly. With sequencing data from diploid HG002 cells with relatively high genomic coverage (average coverage ∼41.7%) on the ONT platform, the N50 can reach more than 1.3 Mb. In addition, using assembled genomes from the K562 single-cell dataset, more complete and accurate sets of insertion events and complex structural variants can be identified. The study opens a new chapter in the *de novo* assembly of single-cell genomes.

Measuring variability between two half-cellular materials uniformly separated from the same single cell can determine whether these variations are caused by true biological heterogeneity or technical noise in single-cell sequencing. Scientists use half-cells from the same cancer cell line, K562, to carry out single-cell microRNA sequencing. By correlating miRNA levels with the expression of predicted target mRNAs in 19 single cells with the same phenotype, it was found that the predicted target is significantly inversely correlated with a large number of miRNA changes, suggesting that microRNA expression variability alone may lead to non-hereditary intercellular heterogeneity (Wang et al., 2019). Using single-cell chromatin accessibility and RNA-seq data from K562 cells, Litzenburger et al. identified the coordination of the cell surface marker CD24 with GATA transcription factor-related chromatin accessibility changes in single cells. GATA/CD24hi cells were found to have the ability to rapidly reestablish heterogeneity within the entire initiating population (Litzenburger et al., 2017).

ESC cells can produce functional cell types by gradually exposing themselves to specific factors to use lineage-specific gene expression procedures. Khateb et al. used RNA-seq, ATAC-seq, Hi-C, etc. to perform single/bulk cell gene expression, histone modification, chromatin conformation, and accessibility transition analysis of ESC pluripotency, acquisition of anterior presomitic mesoderm (aPSM) fate, and further myogenic and neurogenic differentiation, revealing the genomic and transcriptional characteristics and identifying regulatory regions that guide initial Pax7 expression and activation of myogenic and neurogenic procedures (Khateb et al., 2022).

### 3.3 The application to human disease and large animal models

As the most closely related species to humans, primates are the classic animal models for the study of human diseases and can show developmental and important pathological characteristics that are consistent with humans. To better understand the physiological and genetic characteristics of primates, Qu et al. combined single-cell chromatin accessibility and RNA sequencing data from *Macaca fascicularis* to plot a “Monkey Atlas” containing 40 distinct cells from 16 representative organs (Qu et al., 2022). They inferred cell trajectories and intercellular communication, revealed the key molecular features, identified various cell-specific cis-regulatory elements, and constructed organ-specific gene regulatory networks at the single-cell level. In addition, it was found that cynomolgus monkeys had a higher degree of similarity in immune-related gene expression patterns with humans compared to mice. The research provides a valuable resource for animal model research involving non-human primates. Zhang et al. (2020) conducted single-cell transcriptomics involving the aorta and coronary arteries of young and elderly cynomolgus monkeys. The molecular characteristics of specific arteries and eight markers distinguishing the aorta and coronary vascular systems were identified. The analysis found that FOXO3a (a longevity-related transcription factor) was inactivated in the arteries of elderly monkeys. It confirmed that FOXO3a deletion is a key driver of arterial endothelial aging. Arterial aging studies are an important topic in the study of cardiovascular disease. The study provides important clues for how aging affects the cellular and molecular components of the vasculature and causes cardiovascular disease. Both examples show good cases for primate model research and related treatments for human diseases.

Except for non-human primates, pig is a specie that is, closely related to human. Pigs have the advantages of a short growth period, large litter size, a cheap price, and easy editing of genomes (Lunney et al., 2021). They are often used in clinical research and are thought to be the best host species for producing human organs. The complete single-cell landscape of early embryonic development in pigs is constructed. The similarities and differences between pigs and monkeys are analyzed at the single-cell level, which provides new insights for mammalian development and “artificial organs” (Liu T. et al., 2021). Based on single-cell transcriptome sequencing, a cell transcriptome atlas of pigs containing several important tissues/organs was constructed for the first time. It also provided key scientific research resources and a scientific basis for promoting the application of model pigs in the field of biomedicine and xenotransplantation (Wang et al., 2022). The brain tissue of domestic pigs has a multiple brain gyrus structure similar to that of the human brain, which has a high reference value. Zhu et al. (2021) analyzed the single-nucleus transcriptomic data of the domestic pig brain region, and plotted a single-cell transcriptome profiling landscape. This study provided important reference information and effective ideas for using domestic pigs for human neurological disease research or as genetically modified animal models. In addition, there are also many applications in some other organs, such as the eyes (Gautam et al., 2021).

### 3.4 Application and findings in COVID-19 and related animal models

COVID-19 is caused by severe acute respiratory syndrome coronavirus 2 (SARS-CoV-2). It is a single-stranded RNA β-coronavirus that infects human host cells *via* ACE2 and NRP2 receptors. The virus has caused millions of deaths since 2019. A detailed understanding of the dynamics of SARS-CoV-2 infection is important to uncover the viral and host mechanisms that contribute to the pathogenesis of coronavirus disease. Since the outbreak, researchers have analyzed the pathogenesis of SARS-CoV-2 and the immune response of the human body for further vaccine and therapeutic drug development.

The human nasal cavity and alveoli are two parts that are easily accessible to respiratory viruses. Hou (Hou et al., 2021) examined the biomarker spectrum of SARS-CoV-2 serum inflammation in the nasal cavity, bronchoalveolar lavage fluid (BALF), and peripheral blood mononuclear cells (PBMCs) by performing single-cell RNA sequencing. In severe male patients, cell interaction network analysis was conducted to reveal elevated mononuclear cell expression of Toll-like receptor 7 (TLR7) and Bruton tyrosine kinase (BTK). COVID-19 underlying epithelial cell-immune cell interaction and immune vulnerability with increased disease severity and mortality. This study found a high risk for men at different stages of infection, which is sex-biased and susceptible to viral infections. When patients were concurrently infected with the two viruses COVID-19 and influenza A (IAV), single-cell RNA sequencing of peripheral monocytes found that the two viruses synergistically increased pro-inflammatory cytokines and interferons (IFN), and revealed different immune responses after infection with the two viruses (Zhu L. et al., 2020).

There is no specific drug for the SARS-CoV-2 virus, and neutralizing antibodies have the potential to become a specific drug. Thus, Xie and Qin’s rapid identification of SARS-CoV-2 neutralizing antibodies by high-throughput single-cell RNA and VDJ sequencing of antigen-enriched B-cell from 60 convalescent patients (Cao Y et al., 2020). Then, combining with the escape map and the structural analysis, representative antibodies of each epitope of the omicron BA.1 strain were successfully analyzed at the level of a single antibody. The research provided data support for the development of subsequent antibody drugs and broad-spectrum vaccines. Moreover, analysis of human blood immune cells responding to SARS-CoV-2 virus infection would provide insights into the COVID-19 pathological mechanism. Stephenson et al. used CITE-seq to perform single-cell assays on more than 780,000 peripheral blood mononuclear cells from 130 patients with varying degrees of COVID-19. Non-classical monocytes expressing “complement transcripts (CD16 + C1QA/B/C +)” were found to replenish the alveolar macrophage pool in COVID-19. The study highlighted the coordinated immune response of COVID-19 and revealed some discrete cellular components that could be targeted for treatment (Stephenson et al., 2021). Similarly, Unterman et al. also used CITE-seq to detect single-cell transcriptome and cell surface proteins in immunopathologic studies associated with SARS-CoV-2 (Unterman et al., 2022). They explore the dynamic immune response in hospitalized patients with a stable or progressive course of COVID-19. Coordination analysis of gene expression and cell lineage protein markers showed that S100Ahi/HLA-DRlo classic monocytes and activated LAG-3hi T-cell were markers of disease development and revealed the hetero-synchronism of innate and adaptive immune interactions in infection with COVID-19.

During the coronavirus outbreak, animal models have played an important role, and single-cell sequencing has also been used to study the mechanism of virus infection in animal models, which provide an important reference for the treatment of human diseases. Non-human primates are good material when studying COVID-19. Speranza et al. (2021) used single-cell RNA sequencing to demonstrate that SARS-CoV-2 replicates in the lungs of African green monkeys and the population of immune cells in the lungs changes during infection. Sequencing data showed that lung cells are the sites of viral replication and that infiltrative macrophages are responsible for clearing infected cells and cell debris early in infection. The study further deepens our understanding of the dynamics of SARS-CoV-2 infection and the immune response in the host. Similarly, Han et al. used single-cell RNA sequencing techniques to generate single-cell transcriptome profiles of nine monkeys infected with SARS-CoV-2 (Han et al., 2020). It was found that ACE2+ TMPRSS2+ epithelial cells of the lung, kidney, and liver are the targets of SARS-CoV-2. The correlation analysis of the ACE2 receptor found that IDO2 and ANPEP may be potential therapeutic targets, and also revealed the relationships between IL6 and STAT transcription factors.

## 4 The emerging trend of single-cell omics

### 4.1 Trends and challenges for single-cell omics

Studying single-cell multi-omics more comprehensively and systematically will remain as a challenge. On one hand, improve and diversify single-cell sequencing technologies, including increasing the sensitivity and accuracy of scRNA-seq, scDNA-seq and scATAC-seq, lowering the threshold for the detection of mRNA molecules, developing single-cell proteome, single-cell metabolome sequencing techniques, etc. (Svensson et al., 2017). On the other hand, more single-cell omics layers in one cell could be achieved. In the future, four- or five-layer omics data with the whole-transcriptome, spatial transcriptomics, live-cell imaging, chromatin 3D structure, and even expanding to whole omics (capturing all molecules in cells) will be possible (Chappell et al., 2018). Thus, the multi-omic sequencing and functional validation are effective for revealing novel molecular mechanisms, network modules, disease occurrence, and development.

The development of single-cell omics sequencing development has also brought opportunities and challenges to bioinformatics analysis. The first challenge is the ability to process large amounts of different omics data. The increasing demand for data integration in single-cell multi-omics has place addtional computational strain on the available computing resources. It is particularly important to allocate resources and process available data (Ma et al., 2020). Secondly, the comprehensive analysis and processing of multi-omics data is needed, which includes elucidating the statistical and biological characteristics of different omics data, eliminating mixed factors such as batch effects among samples, and also adopting sapiential computational strategies for integrated calculation (2019). Thirdly, it is becoming increasingly important to establish stable benchmarking pipelines for evaluating and testing single-cell multiple omics analysis (Ma et al., 2020). Lastly, online databases will be valuable mining resources for research.

### 4.2 Perspectives for elucidating the pathogenesis of human diseases and related animal models

Causal variations of potential disease risk usually play a role by disturbing the expression of normal genes, and these expressions may be cell type-specific. To date, GWAS has identified millions of genetic variants that affect complex traits and disease risk. Using single-cell omics and multi-omics techniques to depict them would be the main content of human disease research in the post-GWAS era (Gallagher and Chen-Plotkin, 2018). 93% of disease loci associated with human diseases are in intergenic regions or gene deserts, resulting in gene regulatory annotation challenges (Boix et al., 2021). Combining single-cell omic and multi-omics data and machine learning to map and annotate trait variation sites would be effective for studying complex traits and disease pathogenesis. It would be promising when focusing on the genetic essentialities and mocular drivers based on single cell omics data.

With spatial transcriptomes occuring in the technology of the year in 2020 (Nature Methods, 2020), exploring spatial multiomics and spatial dynamics to directly measure as many features as possible in the same cell with spatial resolution (e.g., RNA, DNA, chromatin, protein, and epigenetic modification), as well as examining the tumor microenvironment using spatially sensing single-cell techniques, would be new perspectives for analyzing the pathogenesis of diseases and animal models of diseases in the post-GWAS era (McGuire et al., 2020; Nam et al., 2021). In the COVID-19 period, developing metagenomics would promote our understanding of SARS-CoV-2 pathogenic microorganisms, and with the development of single-cell technology, single-cell microbiome technology will also be an important perspective in the future.
